# The Aging of the Social Mind - Differential Effects on Components of Social Understanding

**DOI:** 10.1038/s41598-017-10669-4

**Published:** 2017-09-08

**Authors:** Andrea M. F. Reiter, Philipp Kanske, Ben Eppinger, Shu-Chen Li

**Affiliations:** 1 Lifespan Developmental Neuroscience, Department of Psychology, Technische Universität Dresden, 01062 Dresden, Germany; 2 Department of Neurology, Max Planck Institute for Human Cognitive and Brain Sciences, 04103 Leipzig, Germany; 3 Department of Social Neuroscience, Max Planck Institute for Human Cognitive and Brain Sciences, 04103 Leipzig, Germany; 4 Institute of Clinical Psychology and Psychotherapy, Department of Psychology, Technische Universität Dresden, 01062 Dresden, Germany; 5 Department of Psychology, Concordia University, Montreal, Canada; 6 PERFORM, Concordia University, Montreal, Canada

## Abstract

Research in younger adults dissociates cognitive from affective facets of social information processing, rather than promoting a monolithic view of social intelligence. An influential theory on adult development suggests differential effects of aging on cognitive and affective functions. However, this dissociation has not been directly tested in the social domain. Employing a newly developed naturalistic paradigm that disentangles facets of the social mind within an individual, we show multi-directionality of age-related differences. Specifically, components of the socio-cognitive route – Theory of Mind and metacognition – are impaired in older relative to younger adults. Nevertheless, these social capacities are still less affected by aging than factual reasoning and metacognition regarding non-social content. Importantly, the socio-affective route is well-functioning, with no decline in empathy and elevated compassion in the elderly. These findings contribute to an integrated theory of age-related change in social functioning and inform interventions tailored to specifically reinstate socio-cognitive skills in old age.

## Introduction

Understanding others is important for successful aging. It has been linked to life satisfaction, wisdom, and lower degrees of loneliness in old age^1, 2^. However, earlier research focused mainly on childhood development and dysfunction of the social mind^3^. Recent studies in younger adults emphasize the importance of understanding subfacets of social understanding^4^. Specifically, social neuroscience research dissociates *cognitive* from *affective* routes of social understanding^5^: the socio-*cognitive* route entails mentalizing and metacognition, whereas the socio-*affective* route encompasses empathy, i.e., the sharing of others’ feelings, and compassion, i.e., a feeling of concern towards others. These routes work independently from each other^5^ and show differential patterns in psychopathology^6^.

In aging research, the socio-emotional selectivity theory^7^ similarly suggests divergent effects of aging on cognitive and emotional functions: whereas cognitive abilities decline, affective functions are considered to stay intact or even increase with old age. However, this pluralistic notion of aging has not been investigated with respect to social understanding. Existing adult developmental studies have examined *either* socio-cognitive *or* socio-affective aspects by using tests of Theory of Mind (ToM) *or* empathy, respectively, which precludes direct comparisons. Consequently, accumulated findings to date are rather equivocal: both the cognitive and affective routes have been shown to be impaired, stable or even enhanced during aging^2, 8–11^. Moreover, social metacognition^12^ as an important facet of social information processing has yet to be studied from an adult developmental perspective.

To close these gaps, this study investigates the effects of aging on component processes of social understanding in a community sample of 55 healthy younger adults and 52 healthy older adults. Specifically, we use a newly developed, naturalistic paradigm, the EmpaToM task^13^. This allows for assessing multiple facets of social understanding (i.e., ToM, social metacognition, empathy, and compassion) within an individual with the same task; thus, making it possible to ascertain potential differential age effects. Extending the socio-emotional selectivity theory, we predict age-related decline in socio-cognitive functions, whereas we expect facets of socio-affective functions to be intact or even improved in old age.

## Results

Results were derived from analyses of ToM, empathy, compassion and metacognition assessed in a video-based paradigm^13^. In each trial, participants viewed either a neutral or an emotionally negative video clip and answered multiple-choice questions about the content of the presented video, either requiring ToM inference or factual reasoning. Participants were also asked to indicate confidence in their answers, to rate how they felt after viewing the video (empathy) and how much compassion they felt towards the protagonist (see methods for further details). These four questions per video allowed us to independently measure ToM (vs. non-social, factual reasoning), metacognition, empathy and compassion in each participant.

### Differential Age Effects in ToM and Factual Reasoning

**Figure 1:**
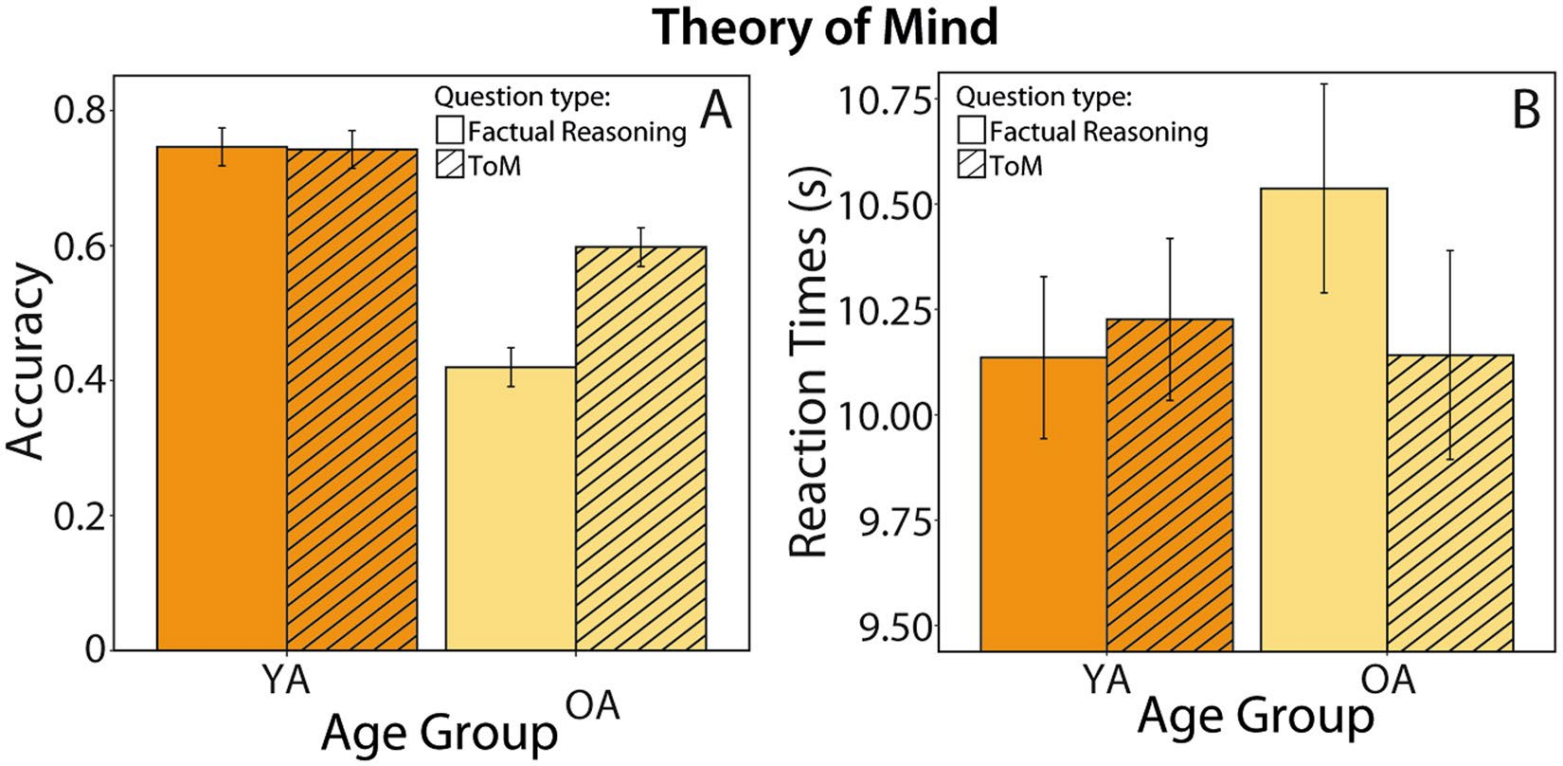
Age differences regarding Theory of Mind. Analysis of accuracy (panel A) and reaction times on correctly answered trials (panel B) consistently revealed a significant interaction of age group and question type (ToM vs. factual reasoning question). Whereas in younger adults no difference between the two question types was apparent, older adults answered ToM questions correctly significantly more often (panel A) and faster (panel B) than factual reasoning questions. Error bars represent 95% confidence interval. Within-subject error bars are adjusted by removing inter-subject variability.

We first analyzed accuracy in the multiple-choice questions which required either ToM inference or factual reasoning. To this end, we used a repeated measures ANOVA with the between-subject factor age group and the within-subject factor question type (ToM vs. factual reasoning/Non-ToM). This analysis revealed a significant main effect of age group (F(1,105) = 126.03 p < 0.001, η_partial_^2^ = 0.55), with the elderly answering less accurately (t(105) = 11.23, p < 0.001), and a significant effect of question type (F(1,105) = 37.84, p < 0.001, η_partial_^2^ = 0.27; Fig. 1a), with a higher accuracy for ToM questions (t(105) = 5.09, p < 0.001).

These main effects were qualified by a significant age group x question type interaction (F(1,105) = 41.21, p < 0.001, η_partial_^2^ = 0.28, Fig. 1a). Post-hoc t-tests revealed that younger adults performed similarly on the factual reasoning and ToM questions (t(54) = 0.19, p > 0.85). In contrast, older adults were significantly more accurate when they had to infer the protagonists’ mental state as compared to factual reasoning (t(51) = 8.80, p < 0.001).

Reaction times (RTs) for correct responses were analyzed using the same ANOVA model. No main effects of age group or question type were found (Fs < 2.72, ps > 0.10). Crucially, however, we found a significant age group x question type interaction (F(1,105) = 4.58, p = 0.04, η_partial_^2^ = 0.04, Fig. 1b). Whereas the RTs of younger adults did not differ as a function of question type (ToM vs. factual reasoning, (t(54) = 0.39, p > 0.25)), older adults responded significantly faster to questions requiring ToM inference than to those requiring factual reasoning (t(51) = 2.61, p = 0.02). Taken together, these results underscore an advantage of social as compared to factual cognition in the elderly.

### Age Differences in Social vs. Non-social Metacognition

**Figure 2:**
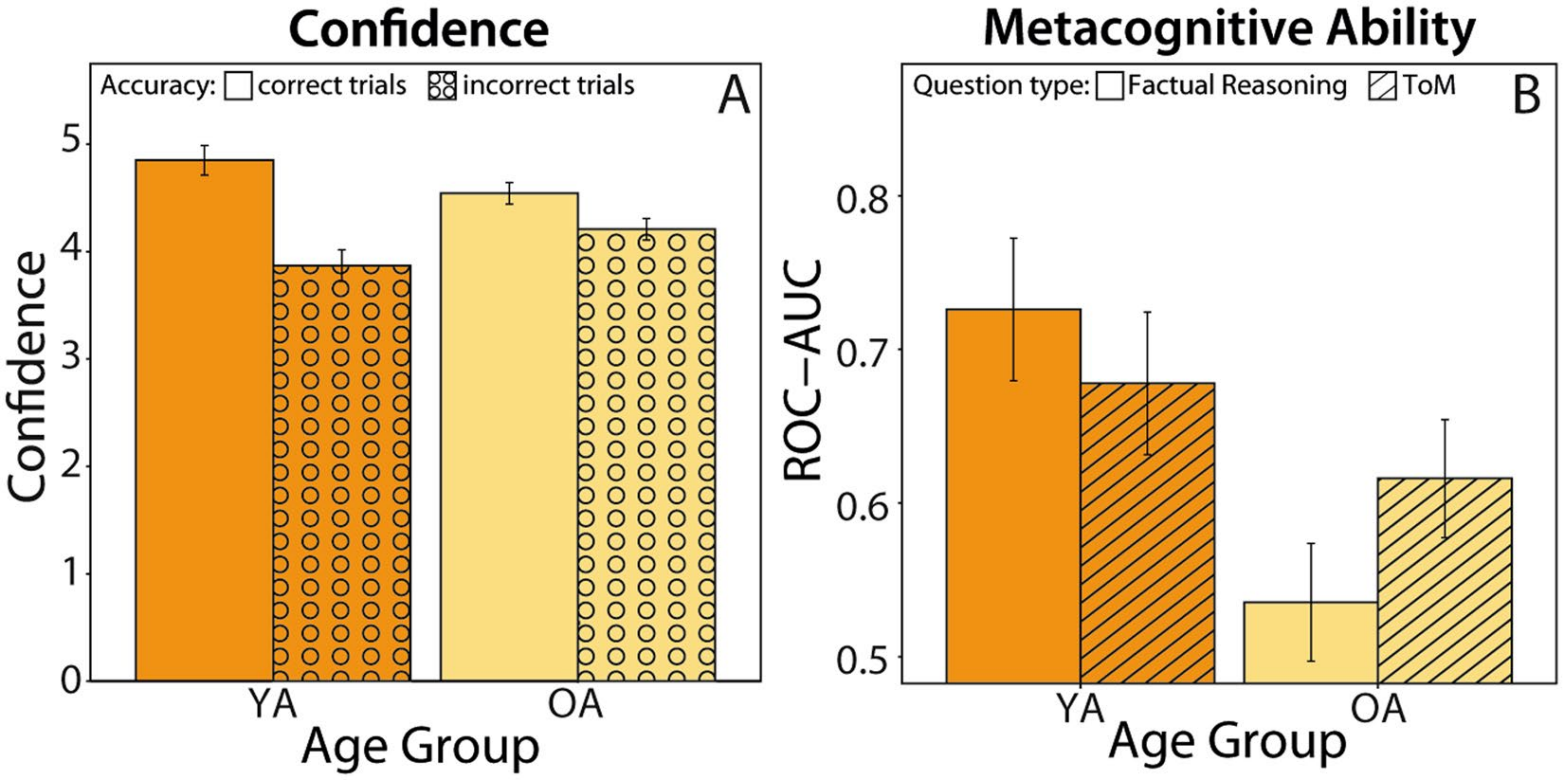
Age Differences regarding Confidence and Metacognitive Ability. Panel A: Confidence ratings for correctly and incorrectly answered questions. Both groups rate their confidence relatively high, also in erroneous trials (mean > 3 on a 1–6 scale). Confidence ratings of older adults are less sensitive to actual response accuracy than those of younger adults. Panel B: A significant interaction effect of age group and question type on metacognitive monitoring ability (indicated by the area under the curve of the receiver operating characteristic) was revealed. In the elderly, social metacognition, the ability to appropriately evaluate one’s own capacity of mentalizing, was less impaired than metacognition regarding factual reasoning capacities. Error bars represent 95% confidence interval. Within-subject error bars are adjusted by removing inter-subject variability.

In each trial, after having answered the multiple-choice question, participants were asked to indicate how confident they were that their given answer was correct. In the next analysis step, we examined age differences in these subjective confidence ratings (while adjusting for individual differences in accuracy as covariates). Overall confidence ratings did not differ significantly between age groups (F(1,104) = 0.83, p > 0.25, η_partial_^2^ = 0.008). When separating correctly answered trials from incorrectly answered trials, we found a difference in the confidence ratings as a function of response accuracy (F(1,105) = 121.47, p < 0.001, η_partial_^2^ = 0.54, Fig. 2a). As Fig. 2a shows, on average the confidence ratings of both groups were significantly lower in incorrect than in correct trials. Interestingly, we also observed a significant age group x response accuracy interaction in the confidence ratings (F = 33.04, p < 0.001, η_partial_^2^ = 0.24). As displayed in Fig. 2a, older adults make relatively more high-confidence errors than younger adults, a pattern that is reminiscent of findings from the research on episodic memory aging^14^. Relatedly, when comparing age differences in measures of metacognition (while adjusting for individual differences in mean accuracy), we found significantly lower metacognitive capacities in the elderly as compared to younger adults (F = 4.84, p = 0.03, η_partial_^2^ = 0.05, Fig. 2b).

In a next analysis step, we were particularly interested in the effects of emotionality and question type on metacognitive capacities in both age groups. Therefore, we tested for effects of question type (ToM vs. factual reasoning) and emotionality, while including mean accuracy values per condition as covariates. The main effects of question type and emotionality as well as their interaction were not significant (Fs < 0.17, ps > 0.25). Interestingly, however, we found a significant interaction of question type with age group (F(1,89) = 4.14, p = 0.045, η_partial_^2^ = 0.04, Fig. 2b). Post-hoc univariate ANOVAs showed that older and younger adults only differed significantly regarding factual metacognition (F(1, 101) = 8.1, p = 0.005, η_partial_^2^ = 0.07), but not with respect to social metacognition (F(1,101) = 0.04, p = 0.844, η_partial_^2^ < 0.001). To summarize, older adults’ confidence ratings discriminate less between correct and incorrect responses than those of younger adults, reflecting age-related impairment in metacognitive monitoring. Importantly, however, this deficit is specific only to non-social, factual contents. The ability to evaluate one’s own capacity of ToM is not significantly impaired in the elderly compared to the young adults.

### No Age Differences in Empathy and Positive Age Differences in Compassion

**Figure 3:**
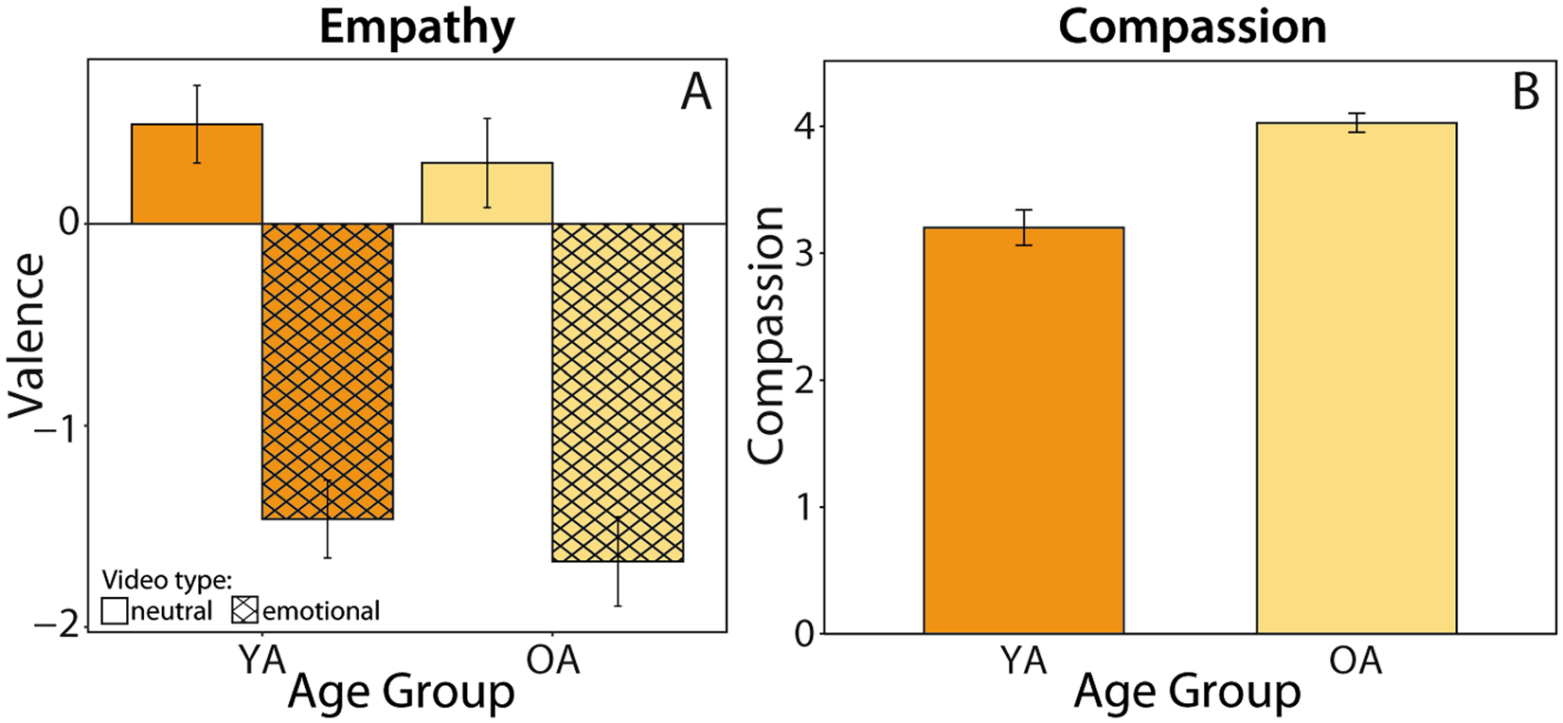
Age differences regarding the affective route of social understanding (Empathy and Compassion). Panel A: empathy ratings showed that in both age groups, emotionally negative videos elicited more negative affect as compared to baseline (i.e., valence ratings after neutral videos). This effect was not significantly moderated by age group, thus no evidence for age group differences regarding empathic responding was observed. Panel B: Older adults show significantly higher compassion than younger adults. Error bars represent 95% confidence interval. Within-subject error bars are adjusted by removing inter-subject variability.

After viewing each video clip, participants rated their own mood. The mood ratings allowed us to assess participants’ empathic responding (i.e., how much their own mood was influenced by the video clip) after videos with an emotionally negative vs. a neutral content. Age differences in these valence ratings were analyzed using a repeated measures ANOVA with age group as the between-subject factor and emotionality (emotionally negative vs. neutral videos) of the video as the within-subject factor. Age differences regarding empathy would emerge as an interaction effect between age group and emotionality. The analysis showed a significant effect of emotionality of the video (F(1,105) = 361.87, p < 0.001, η_partial_^2^ = 0.78), suggesting that emotionally negative videos elicited more empathic responding, that is, more negative affect, in our participants than neutral videos (see Fig. 3a). However, we observed no significant main effect of age group (F(1,105) = 2.45, p = 0.12, η_partial_^2^ = 0.02) and no significant age group x emotionality interaction (F(1,105) = 0.008, p = 0.93, η_partial_^2^ < 0.001). Thus, emotionally negative videos triggered empathic responding in both age groups to a similar extent. Participants were also asked to indicate their compassion towards the video’s protagonist in each trial. Regarding these compassion ratings, an independent t-test showed significant age differences (t(82.74) = 5.20, p < 0.001, Cohen’s d = 0.99); older adults indicated higher ratings of compassion than younger adults (Fig. 3b).

### Independence of Cognitive and Affective Routes of Social Understanding

A recent study using the EmpaToM has found no significant correlations between empathy and ToM measures in a population of younger and middle-aged adults^5^. We were interested in whether the inter-correlation of cognitive and affective social skills may differ as a function of age, as previous studies of cognitive aging found dedifferentiation (higher inter-correlations) between subcomponents of fluid intelligence in older age groups^15^. Thus, for both groups separately, we correlated the ToM measure with the empathy measure. In line with previous findings, neither in younger nor in older adults did we find a significant association between measures of socio-cognitive and socio-affective skills (rs < 0.06, ps > 0.64). Thus, in contrast to cognitive abilities, the sub-facets of the social mind do not exhibit dedifferentiation in old age.

### Differential Effects of Cognition on Age Differences in Socio-cognitive and Socio-affective Abilities

In order to test for potential effects of cognitive abilities on the observed age differences, we repeated the analysis on ToM accuracy, (social) metacognition, empathy, and compassion by statistically adjusting for a unit-weighted composite score of cognitive abilities reflecting fluid intelligence (based on z-scores of working memory, attention, cognitive speed) and the accuracy of the Spot-a-Word Test (as a proxy of verbal intelligence). Adding these covariates in the analyses did not alter the age effects we found on ToM and compassion (ToM: all ps < 0.001, all Fs > 13.07, all η_partial_^2^ > 0.11, compassion: all ps < 0.01, all Fs > 9.00, all η_partial_^2^ > 0.08), nor did they alter the absent interaction effect regarding empathy (all ps > 0.12, all Fs < 2.36, all η_partial_^2^ < 0.02). However, age differences on metacognitive capacities were less pronounced and became non-significant after including the composite score of fluid cognitive abilities as a covariate (F(1,101) = 2.91, p = 0.09, η_partial_^2^ = 0.03). Moreover, the interaction effect of question type on metacognition was non-significant when including the composite measure of fluid cognitive abilities - where older adults scored lower - as a covariate (F(1,87) = 3.26, p = 0.08, η_partial_^2^ = 0.04). The same interaction effect was not significant when adjusting for verbal ability, where older adults scored higher (F(1,100) = 3.52, p = 0.06, η_partial_^2^ = 0.03). These results suggest that whereas general cognitive abilities do not influence age differences regarding ToM, empathy, and compassion, they at least partially account for age effects on metacognition with respect to factual reasoning and social understanding.

## Discussion

Using a new naturalistic, well-validated paradigm, we directly tested the hypothesis that distinct components of social understanding age differentially. Our findings confirm this hypothesis, rather than supporting a uniform pattern of the aging of the social mind.

### Advantage of Socio-affective over Socio-cognitive Functions in Old Age

Here, we assessed both socio-affective and socio-cognitive processes within an individual allowing us to disentangle differential age effects on independent social skills. Altogether, our results clearly speak in favor of an advantage of socio-affective over socio-cognitive functions in old age. First, we show that ToM is significantly less impaired by aging than factual reasoning. Second, given its important role for social interactions^12^, we extend previous research on metacognition focusing on memory functions^14^ to the social domain. Paralleling the results of our ToM analysis, we present evidence that aging is associated with deficits in metacognitive abilities specifically for factual reasoning problems, which do not generalize to social metacognition. Third, by showing intact empathic responding and enhanced compassion in the elderly, we demonstrate preserved or even elevated socio-affective functioning in old age. These findings are in line with the socio-emotional selectivity theory^7^, which suggests that older adults become increasingly selective in focusing more resources on socio-emotional content, rather than on self- and future-oriented goals. The dissociation between cognitive and affective development in old age has been linked to findings on aging-related structural brain changes: Regions critical for affective processing, the ventral medial prefrontal cortex (vmPFC) and the rostral part of the anterior cingulate cortex (ACC), maintain their structural integrity (e.g., cortical thickness) during healthy aging. Contrarily, the more dorsal parts of the ACC and PFC, associated with cognitive executive functions, show more pronounced aging-related decline^16^. Interestingly, one recent investigation using fMRI has suggested that older adults recruit different cortical networks to process empathy than younger adults^11^. Our findings might suggest to investigate the effects of aging on the neural correlates of socio-affective vs. socio-cognitive functions in future studies.

In light of previous findings, we speculate how the differential age-related changes found in this study may relate to age differences in social decision-making. In fact, a recent fMRI study in young adults has suggested independent contributions of socio-cognitive as well as socio-affective functions to pro-social decision behavior^17^. Age-comparative studies on social decision-making have yielded rather mixed results^18^. In some studies using the dictator game, older adults have been reported to exhibit more pro-social behavior^19^, and empathic concern has also been suggested to mediate age-related differences in altruistic choice^9^. In future studies, it would be interesting to address more specifically how age-differences in socio-affective vs. socio-cognitive functions contribute to age differences in social learning and decision-making.

### Methodological Considerations

Previous studies on adult socio-emotional aging yielded mixed results. Most studies lacked carefully designed control conditions^8^ and applied self-report questionnaires, non-naturalistic stimuli, or age-irrelevant scenarios. While the use of naturalistic settings has been deemed important when studying social skills in general^20^, this might even apply more strongly to age-comparative designs: Previous studies have found that the elderly are impaired in decoding emotions from static, isolated inputs^21^. Here, based on well-validated, standardized naturalistic and dynamic stimuli as well as parallel control conditions, we could not detect any age-related deficits regarding socio-affective skills. This is in line with other studies using more naturalistic scenarios, demonstrating that context matters when older adults deal with emotional situations^10, 22^.

It is to be noted that the paradigm employed in this study did not include stimuli of positive emotional valence, i.e., was not designed to assess positive empathy^23^. Positive empathy, i.e. the capacity to share the positive emotions of others, has in fact been argued to be related to key aspects of successful aging, such as improved personal wellbeing and maintained social relationships^23^. Socio-emotional selectivity theory suggests a bias in older adults towards positive stimuli in the memory and attention domain^24^. It would thus be a highly interesting next step to extend our findings on negative empathy towards social understanding of positive emotions

Due to the cross-sectional nature of the study, it remains open whether the age-related differences we observed regarding ToM and compassion are truly age- or rather cohort-related.

### Conclusion and Outlook

Extending socio-emotional selectivity towards the domain of social understanding, our findings contribute to an integrated theory of age-related change in social functioning: We show that socio-cognitive skills are impaired in older adults whereas socio-affective capacities are well-functioning. This allows future work to build on these findings by studying the relationship of both socio-affective and socio-cognitive capacities with physical, mental and social well-being in older age. In younger adults, the EmpaToM has already been successfully applied in a training study^25^, which opens a promising avenue for studying plasticity of distinct components of social understanding in a longitudinal fashion. The malleability of social skills has been investigated in psychopathology^26^ and in healthy younger to middle-aged adults^25^ but not in older adults. Our findings inform the design of such training programs for older adults regarding specificity: they suggest prioritizing socio-cognitive over socio-affective training in older adults. An intriguing question is whether the impairment in ToM, as found here for older adults, can be improved via specific training programs. A further interesting possibility is that cognitive information processing – be it in the social or the non-social domain – may be facilitated in healthy older adults by encouraging them to use their relatively intact socio-affective abilities.

## Methods

### Participants

Younger adults (YA, range = 18–30 years; n = 57) and older adults (OA; range = 65–80 years, n = 65) participated in the study. Participants were recruited via the database of the Lifespan Developmental Neuroscience Lab at TU Dresden. Only participants fluent in German were invited. A total of 15 participants were excluded from the final data analysis due to the following a priori exclusion criteria: dementia screening based on the Montreal Cognitive Assessment (MOCA^27^ with a score below 25 points (n = 6 OA)), self-report of a current psychiatric condition or of being in psychotherapeutic or psychopharmacological treatment (n = 2 YA, n = 2 OA), any present or past neurological conditions (n = 3 OA, stroke or Morbus Parkinson), below chance level (<0.33) performance (n = 2 OA). Altogether, the final effective sample included 55 younger adults (mean age = 24.29 years, SD = 3.09 years, 31 female) and 52 older adults (mean age = 72.08 years, SD = 3.76 years, 30 female). We aimed for an effective sample size of around 55 participants per age group based on meta-analytic reviews on affective processing^21^ and ToM^8^. These meta-analyses suggested overall medium effect sizes of aging on socio-affective and socio-cognitive processes. Power analyses, based on such expected medium effect sizes (f = 0.25), a two-tailed α = 0.05, and a minimum power of 1−β = 0.80, verified that the final sample size was appropriate to detect between group effects (minimum total sample size: n = 98), within-subject effects as well as the interaction of both (minimum total sample size: n = 34).

Participants were compensated with 8.50 Euro/hour for participation. Ethical approval in accordance with the Helsinki declaration was granted by the TU Dresden ethics committee. All participants provided written informed consent prior to participation.

### Assessing sub-components of social understanding in one paradigm

**Figure 4:**
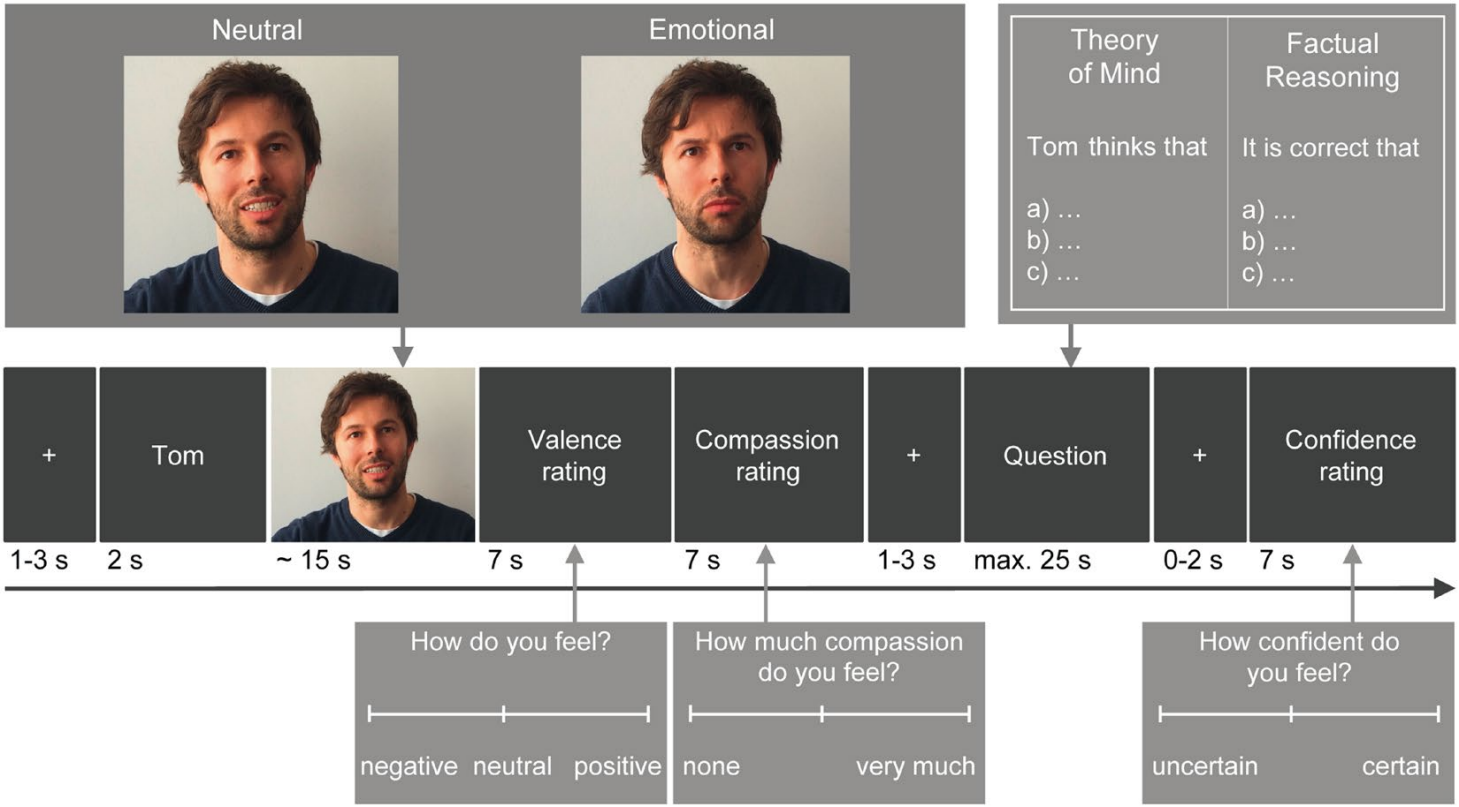
Exemplary Trial Sequence of the EmpaToM Task. In each trial, the participant is presented with an emotionally negative vs. neutral video sequence of a male or a female person speaking about autobiographical experiences (factor emotionality). This video requires either ToM inference or factual reasoning (factor question type). After having seen the video, participants rate their own affect and compassion towards the protagonist in the video. Valence ratings after emotionally negative vs. neutral videos are used as a measure of empathy, that is, how much the participant shares the negative feelings expressed by the video’s protagonist. Lastly, the participants answer content-based multiple-choice questions requiring ToM inference or factual reasoning and rate their own confidence in their answers, allowing us to assess metacognitive monitoring ability regarding own social (i.e., mentalizing) vs. non-social (factual reasoning) performance. Note that the exemplary images depicted in this figure are not based on the original video stimuli used in the EmpaToM task but, for illustration purposes, have been replaced with re-staged images due to license restrictions.

We used the EmpaToM task^13^, a newly developed paradigm to measure ToM, social metacognition, empathy, and compassion, which had been previously validated in young to middle-aged adults. In this naturalistic but well-controlled task (see Fig. 4), participants were presented with a ~15-second video clip in each trial during which a male or a female speaks about an autobiographical experience. The described scenario was either neutral (e.g., selling items on eBay) or negatively emotional (e.g., one’s own sister suffering from bowel cancer) in content. After viewing the video, participants indicated on a continuous valence scale (from negative to positive) how they felt, allowing us to assess empathic responding. Subsequently, they also rated on a continuous scale (from none to very much) how much compassion they felt for the person they had just seen in the video. Lastly, inference on video content was tested in a multiple-choice question with three possible response options, only one of which was correct. Crucially, this question could either require ToM inference (i.e., asking “The person thinks that…”) or factual reasoning (i.e., asking “It is correct that…”). After having responded to the inference question, participants were asked to indicate how confident they felt about the accuracy of their answers. This latter question enables assessment of metacognitive abilities in the social and non-social domain as well as confidence in one’s own accuracy. A total of 48 videos were presented, rendering 12 videos per condition (valence by question type). The task was instructed and carried out in exactly the same manner as validated in a prior large-scale study^13^, but with an extended maximum response time windows (7 s for the ratings and 25 s for the inference questions about video content) to accommodate findings from a pilot study indicating that older adults needed more time to answer the questions than provided in the original version (see Fig. 4 for more details; for exemplary video stories and questions see supplement of ref. 13).

### Cognitive and trait measures

Participants completed a standardized battery of cognitive tests (cf. refs 15 and 28 for similiar batteries of cognitive tests), assessing attention (Trail Making Test A, TMT A), cognitive speed (Identical Pictures Test and Digit Symbol Substitution Test(DSST)), complex attention/executive functioning (Trail Making Test B, TMT B), working memory (Digit Span forward and backward), and verbal ability (Spot a Word test). See Table 1 for a summary of these measures for both groups. In sum, OA scored significantly lower in most measures of cognitive mechanics but higher on the verbal abilities than YA. Altogether, our sample seems to be largely comparable to other population-based lifespan samples^15^.

**Table 1:** Cognitive and trait affect measures in younger and older adults.

	Older Adults	Young Adults	Test statistic
Cognitive Measurements*			
Working Memory (Forward Digit Span)	9.04 ± 1.79	9.49 ± 1.68	*t* = 1.35, *p* = 0.18
Working Memory (Backward Digit Span)	6.15 ± 1.66	7.47 ± 2.04	*t* = 3.68*, p* < 0.001
Cognitive Speed (Identical Pictures Test)	11.51 ± 12.22	17.27 ± 14.11	*t* = 2.25, *p* = 0.03
Cognitive speed (DSST)	60.67 ± 13.35	85.53 ± 6.07	*t* = 4.81, *p* < 0.001
Complex attention (TMT B, time in sec)	88.94 ± 36.29	50.54 ± 15.48	*t* = 9.07, *p* < 0.001
Attention (TMT A, time in sec)	39.54 ± 14.31	22.88 ± 6.40	*t* = 7.76, *p* < 0.001
Verbal/crystallized IQ (Spot a word test)	40.18 ± 9.91	21.00 ± 11.15	*t* = 9.37, *p* < 0.001
Trait Affect*			
PANAS positive mood	34.25 ± 5.23	34.70 ± 6.12	*t* = 0.69, *p* > 0.25
PANAS negative mood	15.96 ± 4.20	19.72 ± 6.12	*t* = 3.69*, p* < 0.001

*Only participants included in the reported final analysis of the data.

Participants also filled out the German version of the PANAS negative and positive mood trait questionnaire^29^, compare Table 1. OA scored lower on negative trait affect than YA, whereas there was no age difference regarding positive affect (Table 1), which is in line with socio-emotional selectivity theory^7^.

### Data analysis

We derived measures for ToM, metacognition, empathy, and compassion in the same manner as has been validated in previous large-scale studies^5, 13^. All analyses were performed using MATLAB and Statistics Toolbox, R2016a (MathWorks, Inc., Natick, Massachusetts, United States), R (R core team, 2016)^30^ and IBM SPSS statistics (IBM Corp, Armonk, NY, United States).

Age differences in the capacity of ToM were analyzed using a repeated measures ANOVA on error rates and RTs, with question type as a within-subject factor and age group as a between-subject factor. Metacognitive ability was determined by computing the area under the curve (AUC) of the receiver operating characteristic curve (ROC) for each participant in each condition. This was done by using the participants’ trial-by-trial accuracy as the predicted state variable as well as their confidence ratings as predictors to the MATLAB function *perfcurve*. The function derives true positive and false positive rates describing a non-parametric, trapezoidal approximation to determine the AUC. Higher ROC-AUC scores indicate higher levels of metacognitive ability. Social metacognition was defined as metacognitive ability in ToM as compared to factual reasoning trials. Confidence ratings and metacognitive ability scores were adjusted for individual differences in accuracy in all analyses.

Age differences regarding empathy (valence ratings) were analyzed using a repeated measures ANOVA with emotionality (negative vs. neutral videos) as a within-subject factor and age group as a between-subject factor. Empathic responding was operationalized as the difference in valence ratings after neutral (i.e., participant’s baseline affect) and emotionally negative videos. Thus, group differences regarding empathy would emerge as an interaction effect between age group and emotionality. Age differences regarding compassion were analyzed using an independent t-test on the compassion ratings.
